# Alpha-Tocopherol prevents esophageal squamous cell carcinoma by modulating PPARγ-Akt signaling pathway at the early stage of carcinogenesis

**DOI:** 10.18632/oncotarget.21437

**Published:** 2017-09-30

**Authors:** Miao Xu, Hui Yang, Qiannan Zhang, Ping Lu, Yongquan Feng, Xue Geng, Lishi Zhang, Xudong Jia

**Affiliations:** ^1^ West China School of Public Health, Sichuan University, Chengdu, China; ^2^ Key Laboratory of Food Safety Risk Assessment of Ministry of Health, National Center for Food Safety Risk Assessment, Beijing, China; ^3^ Beijing University of Agriculture, Beijing, China

**Keywords:** vitamin E, tocopherol, esophageal cancer, Akt, PPARγ

## Abstract

The poor prognosis of esophageal squamous cell carcinoma (ESCC) emphasizes the urgent need to better understand the carcinogenesis and develop prevention strategies. Previous studies have highlighted the potential of using Vitamin E (tocopherols) for cancer chemoprevention, but the preventive activity of α-Tocopherol against ESCC remains to be elucidated. Our data showed that early-stage supplementation with α-Tocopherol significantly prevented esophageal carcinogenesis induced by *N*-nitrosomethylbenzylamine (NMBA) in ESCC rat model. In the Het-1A cell model, α-Tocopherol markedly suppressed cell proliferation, promoted cell cycle G2-phase arrest and increased apoptosis. Gene microarray and proteins array analysis indicated that Akt signaling was a potential target for α-Tocopherol. We further demonstrated that α-Tocopherol increased the expression of PPARγ and its downstream tumor suppressor PTEN. Knockdown of PPARγ activated Akt signaling transduction, whereas this process was attenuated by the presence of α-Tocopherol and PPARγ agonist Rosiglitazone. In contrast, the effect of α-Tocopherol on Akt inhibition was not observed in established tumors, neither in cancerous cell lines which constitutively expressed higher levels of PPARγ. These results were closely correlated with the ineffectiveness of α-Tocopherol in the late stage of ESCC carcinogenesis. Taken together, our study suggested that α-Tocopherol may serve as a PPARγ agonist for the chemoprevention of esophageal cancer.

## INTRODUCTION

Esophageal squamous cell carcinoma (ESCC), which is the predominant histopathological form of esophageal cancer, often results in poor prognosis due to the limited clinical approaches available for early diagnosis and therapeutic treatment [1, 2]. As the eighth most common malignancy worldwide, ESCC causes swallowing difficulty, pain, malnutrition, and lower life quality in patients, with the five-year survival rate around only 20% [3, 4]. The high prevalence and poor outcome of ESCC strongly emphasize the urgent need to better understand esophageal carcinogenesis and develop prevention strategies against this disease.

Previous studies have highlighted the potential of using Vitamin E (α-Tocopherol) for cancer chemoprevention [5]. Epidemiology studies in Linxian, an area in northern China with high incidence of ESCC, suggested that dietary deficiency of α-Tocopherol and selenium was associated with higher risk of ESCC and gastric cardia cancer [6, 7]. The nutritive intervention trial conducted in this area revealed protective activity of the combination of α-Tocopherol, selenium and β-carotene against gastric cardia cancer [8]. However, several large-scale intervention studies indicated that α-Tocopherol might be ineffective in the prevention of prostate cancer, lung cancer, and breast cancer [9–13]. Furthermore, some recent studies suggested that vitamin E and analog promoted lung cancer progression [14] and melanoma metastasis in mice [15]. The interpretation of these results may vary and the cancer preventive activities of tocopherols are still unclear [5, 16].

Interestingly, the prevention of ESCC by combined nutrients in Linxian trial was demonstrated after a follow up for 10 years and only in subjects who entered the trial at age <55 years old [17]. We speculated that the timing of intervention might significantly affect the efficacy of α-Tocopherol/selenium for ESCC prevention. To validate this hypothesis, we have demonstrated that a diet that was low in α-Tocopherol and selenium enhanced *N*-nitrosomethylbenzylamine (NMBA)-induced esophageal carcinogenesis in rats, and this process was attenuated by dietary supplementation with α-Tocopherol and selenium [18]. Importantly, more pronounced inhibition was observed in the combined supplementation at the early state of carcinogenesis [18, 19]. Though the efficacy of combination of α-Tocopherol and selenium in ESCC prevention has been suggested in human and animal studies, mechanism underlying the chemopreventive activity of α-Tocopherol has not been completely elucidated.

In this study, we are focusing our esophageal cancer prevention study with α-Tocopherol on the early stage of ESCC carcinogenesis. We compared the cancer preventive effects of α-Tocopherol between initiation-stage and post-initiation stage supplementation in an NMBA-induced ESCC rat model. The function of α-Tocopherol on cell proliferation, cell cycle and apoptosis was analyzed with Het-1A cell model. Transcriptome profiling identified that PI3K-Akt signaling pathway was suppressed by α-Tocopherol. Akt inhibition by α-Tocopherol was found to be closely related with the expression of PPARγ. These new findings were further validated by *in vitro* studies with normal and cancerous cell lines, as well as in subcutaneous tumor models with athymic mice.

## RESULTS

### Esophageal tumorigenesis was suppressed by dietary supplementation with α-Tocopherol at the initiation stage and post-initiation stage

**Figure 1:**
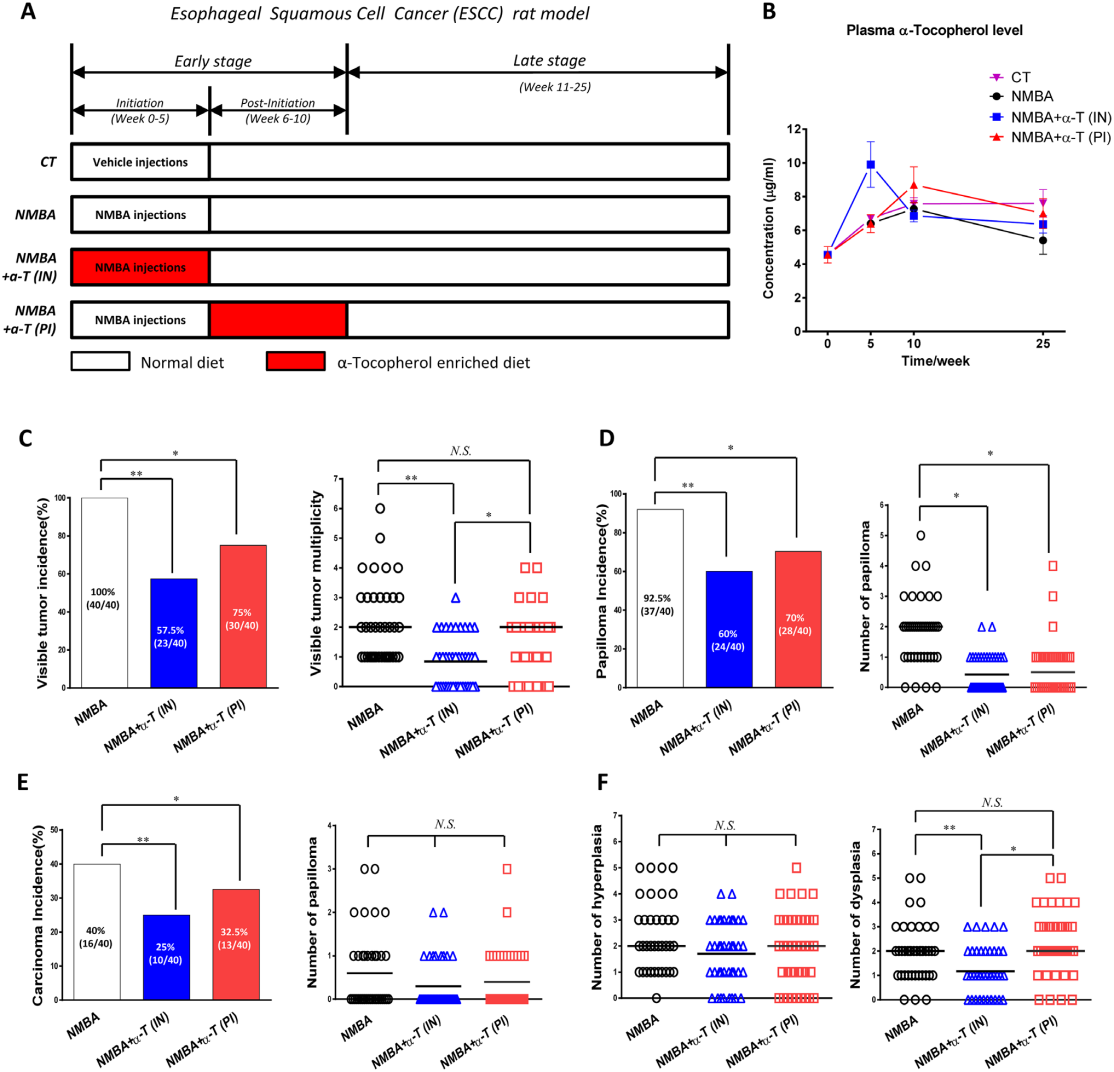
Esophageal tumorigenesis was suppressed by dietary supplementation with α-Tocopherol at the initiation stage and post-initiation stage in ESCC rat model **(A)** Study design with ESCC rat model. **(B)** Monitor of plasma levels of α-Tocopherol using HPLC. **(C)** Incidence and multiplicity of visible tumors in rat esophagus. **(D)** Incidence and number of microscopic papilloma. **(E)** Incidence and number of carcinoma. **(F)** Number of histopathological hyperplasia and dysplasia. “α-T(IN)” represents “supplementation at initiation stage” while “α-T(PI)” represents “supplementation at post-initiation stage”. Data are shown as mean ± standard deviation, 40 animals of each group were analyzed (n=40). ^*^ signifies p<0.05; ^**^, p<0.01; N.S. not significant.

To clarify the chemopreventive activity of α-Tocopherol, we determined the NMBA-induced tumorigenesis in ESCC rat model with dietary α-Tocopherol supplementation respectively at the initiation stage (Week 0-5) and post-initiation stage (Week 6-10). Nutrition monitoring indicated that plasma level of α-Tocopherol was significantly elevated by the dietary supplementation at specific period when compared to the control animals, and the α-Tocopherol levels promptly descended as the intervention withdrew (Figure 1B). Tumor assay at the end of ESCC animal experiment showed that α-Tocopherol dramatically decreased tumor incidence (to 57.5% and 75%, respectively) in rat esophagus (Figure 1C). Interestingly, the initiation-stage supplementation exhibited more efficacy to decrease the multiplicity of visible tumors than the post-initiation supplementation. In parallel, pathological examination also showed that more inhibition of papilloma and carcinoma incidences was induced by the initiation-stage supplementation, though the differences were not statistically significant between these two groups (Figure 1D and 1E). As for the microscopic lesions, no differences were observed on the number of hyperplasia; however, the generation of dysplasia was markedly suppressed by the initiation-stage supplementation, which was significantly less than that in post-initiation supplementation (Figure 1F). Taken together, these data suggest that dietary supplementation with α-Tocopherol at the early stage of ESCC can dramatically inhibit NMBA-induced esophageal carcinogenesis in rat model, and the initiation-stage intervention is more effective than the later-stage intervention. Though only male rats were used in this model, α-Tocopherol is expected to be also protective in female animals due to the similar biological feature for NMBA activation and α-Tocopherol metabolism in male and female animals.

### α-Tocopherol inhibited cell proliferation at early stage of ESCC but was not effective in established tumors

**Figure 2:**
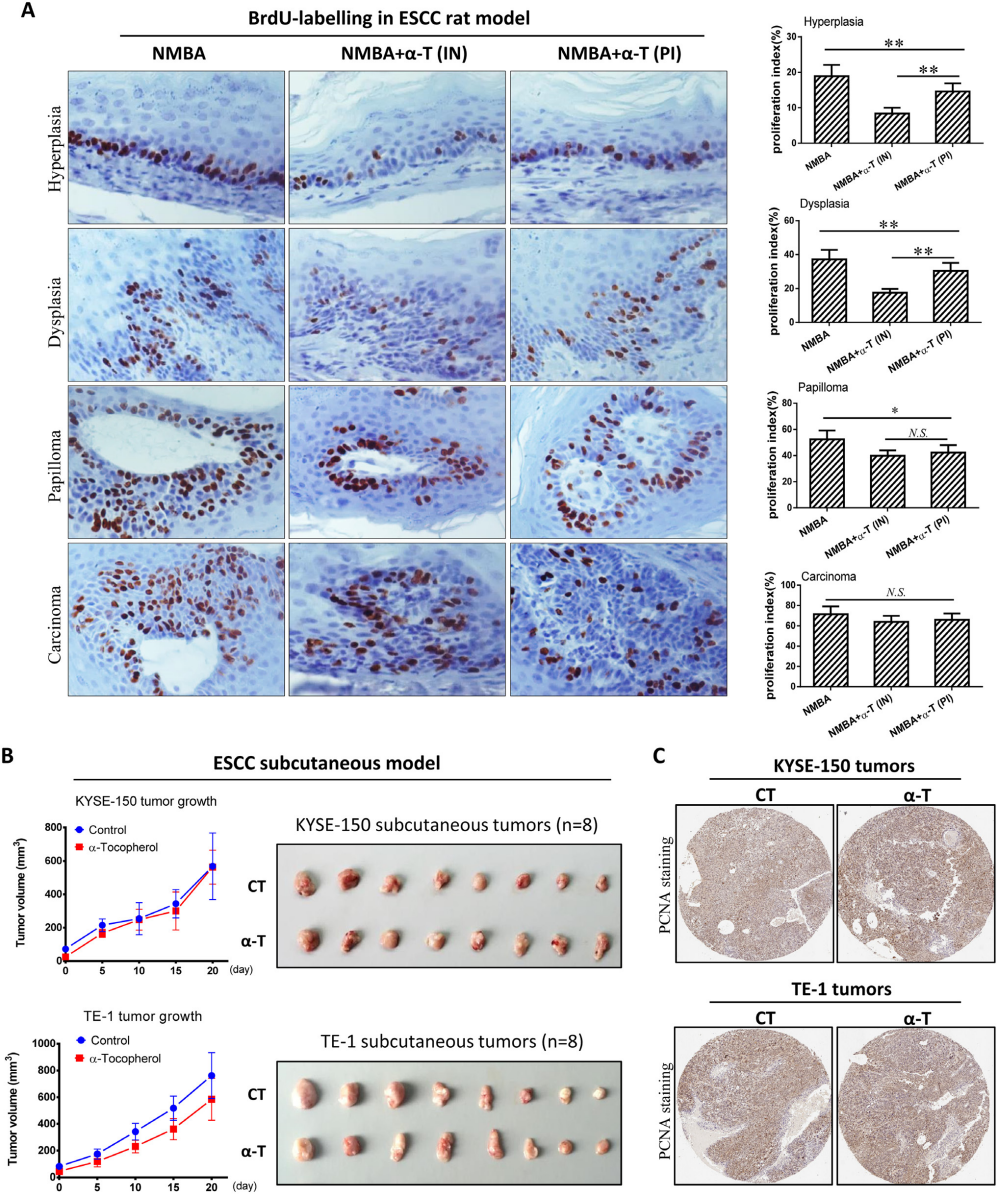
α-Tocopherol inhibited cell proliferation at early stage of ESCC but was not effective in established tumors **(A)** Cell proliferation in rat esophageal epithelium determined by immunostaining of BrdU. Proliferation index of each lesion was calculated as the number of BrdU-positive cells divided by the total number of epithelial cells. Esophageal tissues were randomly selected from 12 rats in each group and used for analysis (n=12). **(B)** The tumor growth curve in subcutaneous models generated with esophageal cancer KYSE-150 and TE-1 cells. **(C)** IHC staining of PCNA in subcutaneous tumors. Data are shown as mean ± standard deviation, ^*^ signifies p<0.05; ^**^, p<0.01; N.S. not significant.

To further investigate the effect of α-Tocopherol on ESCC pathological progression, we determined cell proliferation in different categories of pathological lesions in rat esophagus with *in situ* BrdU immunostaining. As the esophageal epithelium evolving from hyperplasia to carcinoma, the proportion of proliferating cells significantly increased. Supplementation with α-Tocopherol dramatically repressed cell proliferation in hyperplasia, dysplasia and papilloma; however, no significant change was observed in carcinoma. Interestingly, α-Tocopherol supplementation at the initiation-stage showed more marked inhibition of cell proliferation than the supplementation at post-initiation stage, but no difference was observed in the advanced lesions (i.e. papilloma and carcinoma) (Figure 2A). Therefore, we speculated that the inhibitory effect of α-Tocopherol on cell proliferation was more profound in the early-stage pre-malignant lesions than in established tumors.

In addition, we tested the effect of α-Tocopherol on the growth of subcutaneous KYSE-150 and TE-1 tumors with nude mice model. As expected, α-Tocopherol did not significantly suppress the growth of subcutaneous tumors established with KYSE-150 and TE-1 cancerous cells (Figure 2B). The tumor volumes were not reduced by dietary supplementation with α-Tocopherol. Furthermore, the expression of PCNA in tumors was not significantly decreased by α-Tocopherol, either (Figure 2C). Altogether, α-Tocopherol is capable of inhibiting cell proliferation at the early stage of ESCC but not effective in established tumors.

### α-Tocopherol induced cell cycle G2 arrest and apoptosis in NMBA-treated normal esophageal epithelial cells but was not effective in ESCC cell lines

**Figure 3:**
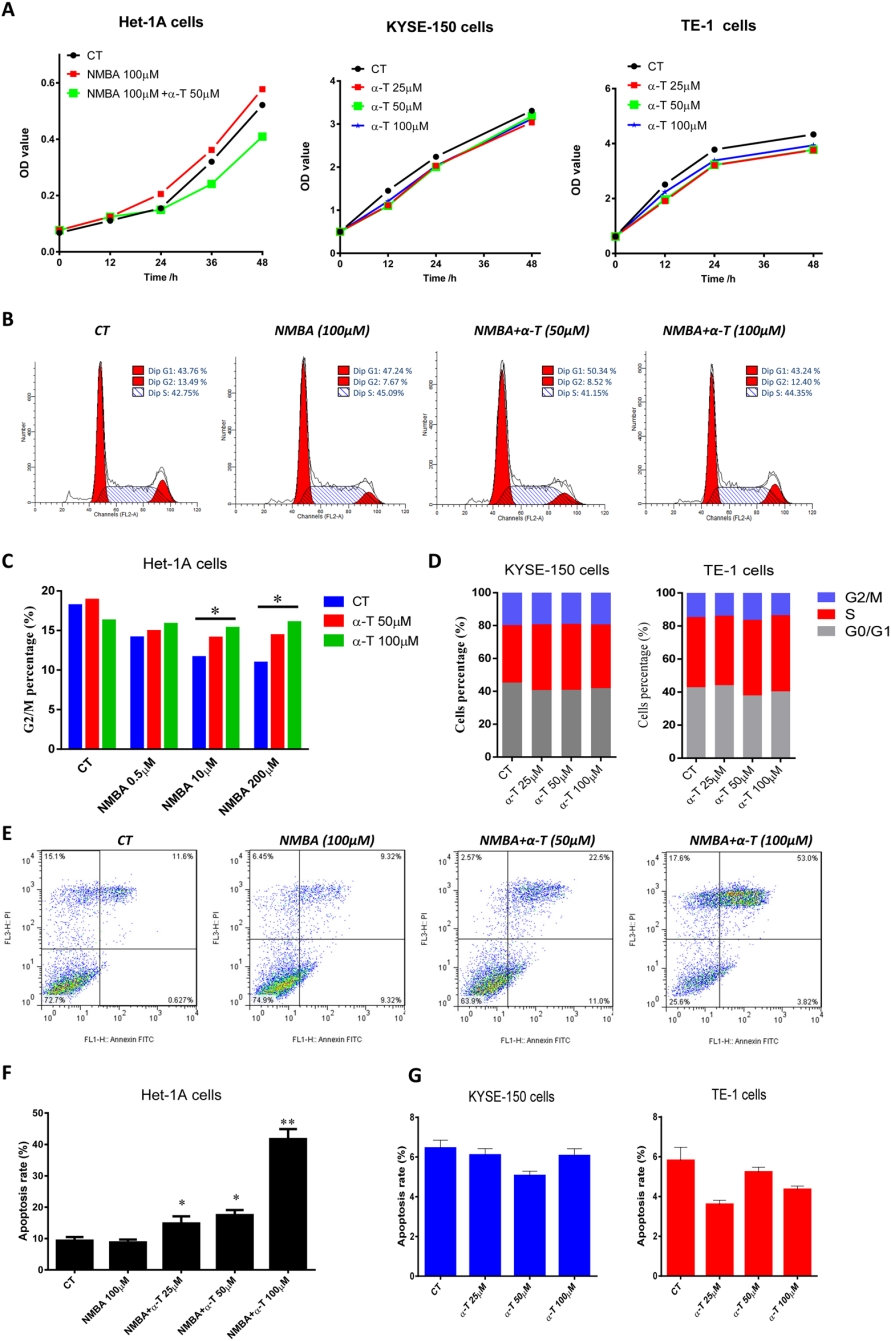
α-Tocopherol induced cell cycle G2 arrest and apoptosis in NMBA-treated normal esophageal epithelial cells but was not effective in ESCC cell lines **(A)** Cell proliferation determined with CCK-8. **(B)** Cell cycle determined with flow cytometry. Het-1A cells were treated with NMBA (100 μM) with or without α-Tocopherol (50, 100 μM) for 72h. **(C)** Cell proportion of G2/M-phase. **(D)** Cell cycle analysis of KYSE-150 and TE-1 cells. KYSE-150 and TE-1 cells were treated with α-Tocopherol (25, 50, 100 μM) for 72h. **(E)** Cell apoptosis determined with Annexin/PI by flow cytometry. Het-1A cells were treated with NMBA (100 μM) with or withoutα-Tocopherol (25, 50, 100 μM) for 72h. **(F)** Apoptotic rate of Het-1A cells. **(G)** Apoptotic rate of KYES-150 and TE-1 cells. KYSE-150 and TE-1 cells were treated with α-Tocopherol (25, 50, 100 μM) for 72h. Data are shown as mean ± standard deviation. ^*^ signifies p<0.05; ^**^, p<0.01; compared with the NMBA group.

To confirm the inhibitory effect of α-Tocopherol on cell proliferation at the early stage especially initiation stage of ESCC, we investigated the impact of α-Tocopherol on cell cycle and apoptosis with cell models. In agreement with the results from *in vivo* studies, α-Tocopherol significantly suppressed cell proliferation in the NMBA-treated human normal esophageal epithelium Het-1A cells, but no significant repression was observed in the human cancerous KYSE-150 and TE-1 cells (Figure 3A).

In the Het-1A cell model, we found that the proportion of G2/M phase cells was gradually decreased by NMBA (0.5, 10 and 200 μM) in a dose-dependent manner, but the NMBA-induced reduction in G2 phase was effectively equilibrated by the presence of α-Tocopherol (50 and 100 μM) (Figure 3B and 3C), suggesting that α-Tocopherol induces G2-phase arrest in the Het-1A cells. In contrast, cell cycle was not significantly altered by α-Tocopherol (25, 50 and 100 μM) in KYSE-150 and TE-1 cells (Figure 3D). Similarly, the cell apoptotic rate was dose-dependently increased by α-Tocopherol in the NMBA-treated Het-1A cell model (Figure 3E and 3F). However, no significant change of apoptosis by α-Tocopherol was observed in the KYSE-150 and TE-1 cells (Figure 3G). These data indicate that α-Tocopherol inhibits cell proliferation of NMBA-treated Het-1A cells by inducing G2-phase arrest as well as by promoting apoptosis.

### α-Tocopherol inhibited ESCC initiation by targeting Akt signaling pathway *in vitro* and *in vivo*

**Figure 4:**
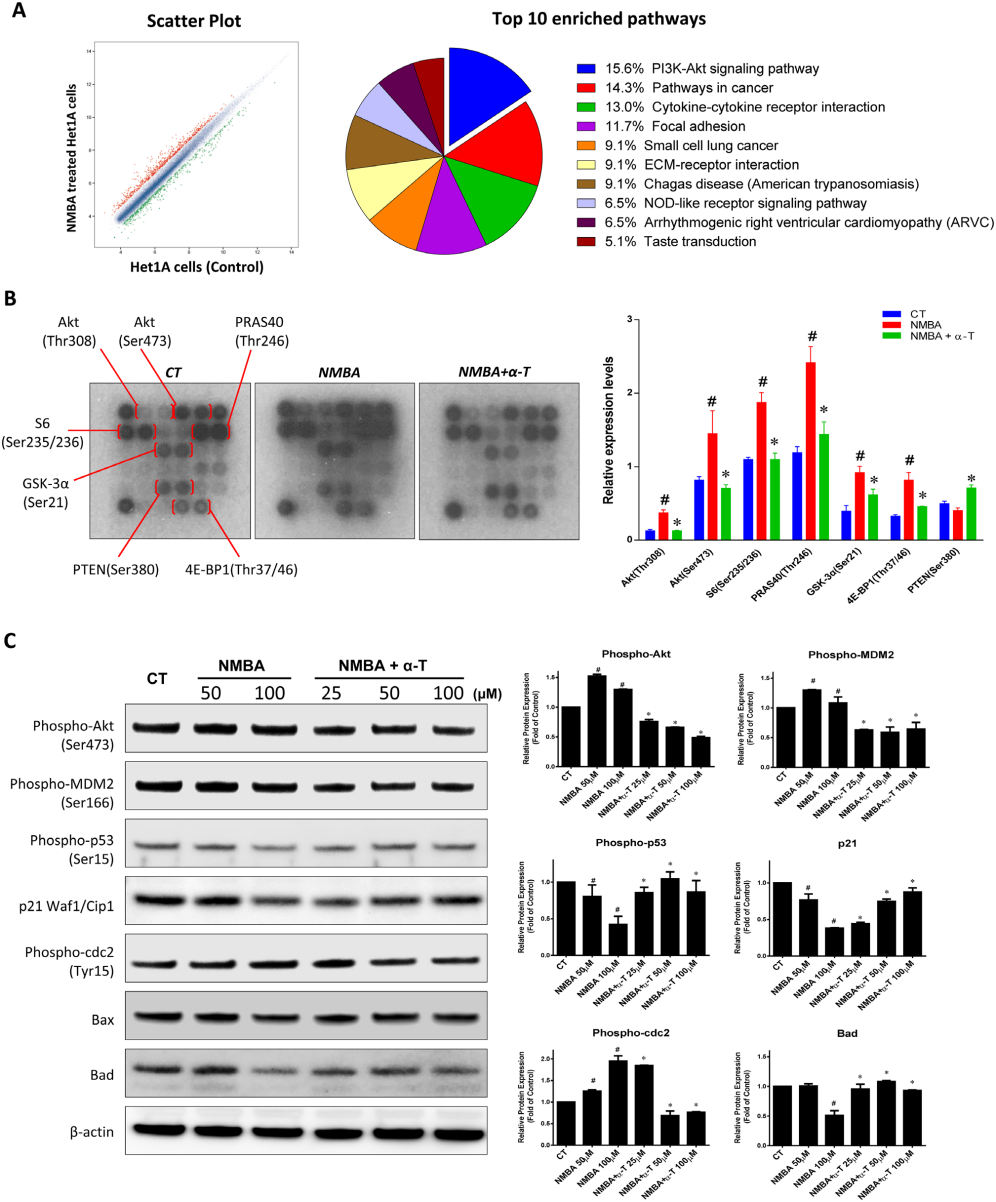
α-Tocopherol inhibited ESCC initiation by targeting Akt signaling pathway in Het-1A cell model **(A)** Scatter plots and pathway enrichment by transcriptome microarray. Het-1A cells were treated with NMBA (100 μM) for 5 weeks. Total RNA isolated for transcriptomic profiling in comparison with untreated cells. (Red dots, up-regulated genes; green dots, down-regulated genes; Pie chart, proportion of pathway-related genes.) **(B)** Proteomic array analysis on Akt signaling pathway. Het-1A cells were treated with NMBA (100 μM) with or without α-Tocopherol (50 μM) for 72h. **(C)** Western blotting of Akt signaling pathway in Het-1A cells. Het-1A cells were treated with NMBA (50, 100 μM) with or without α-Tocopherol (25, 50, 100 μM) for 24h. Data are shown as mean ± standard deviation. ^#^, p<0.05 compared with the control group; ^*^, p<0.05 compared with the NMBA group.

To elucidate the underlying mechanisms of ESCC inhibition by α-Tocopherol at the initiation stage, we carried out transcriptome profiling with Het-1A cell model. When the normal Het-1A cells underwent the 5-week NMBA-treatment, 486 genes (red dots) were up-regulated and 639 genes (green dots) down-regulated (Figure 4A). Based on the differentially expressed genes, top ten enriched pathways were identified. The differently-expressed genes for the enriched pathways were summarized in Supplementary Table 1. Particularly, “PI3K-Akt signaling pathway” and “Pathways in cancer” as the top two pathways accounted for almost 30% of total included genes (Figure 4A and Supplementary Table 1). It has been known that dysregulation of the PI3K-Akt pathway is implicated in a number of human diseases including cancer, diabetes, cardiovascular diseases and neurological diseases. Thus, we speculated that the Akt signaling might be a potential target for α-Tocopherol to block ESCC initiation. With the Akt Signaling Array assay, we observed that NMBA treatment significantly increased the phosphorylation of Akt (Ser 473/Thr 308), S6 ribosomal protein (Ser 235/236), PRAS40 (Thr246), GSK-3α (Ser21), and 4E-BP1 (Thr37/46) (Figure 4B). Furthermore, the activation of these proteins was dramatically inhibited by the presence of α-Tocopherol, suggesting that α-Tocopherol significantly attenuated the activation of Akt signaling pathway during ESCC initiation induced by NMBA.

In addition, we further investigated the impact of α-Tocopherol on the downstream molecules of Akt that governing cell proliferation, survival and apoptosis with Het-1A cell model. Firstly, the blockade of Akt activation by α-Tocopherol was confirmed at various dose levels (25, 50, 100 μM) (Figure 4C). Akt-mediated phosphorylation of mdm2 at Ser166 was also markedly decreased by α-Tocopherol, which prohibited the degradation of the tumor suppressor p53. In agreement, expression of the CDK inhibitor p21 was significantly repressed by NMBA and then dose-dependently restored by α-Tocopherol. Inversely, the phosphorylation of cdc2 that promotes cell cycle G2/M transition was increased by NMBA whereas suppressed by α-Tocopherol, which is in line with the effect of α-Tocopherol on cell cycle arrest at G2-phase. Finally, the expression of pro-apoptotic proteins Bad and Bax which are suppressed by Akt activation were consistently inhibited by NMBA treatment and effectively rescued by α-Tocopherol (Figure 4C).

**Figure 5:**
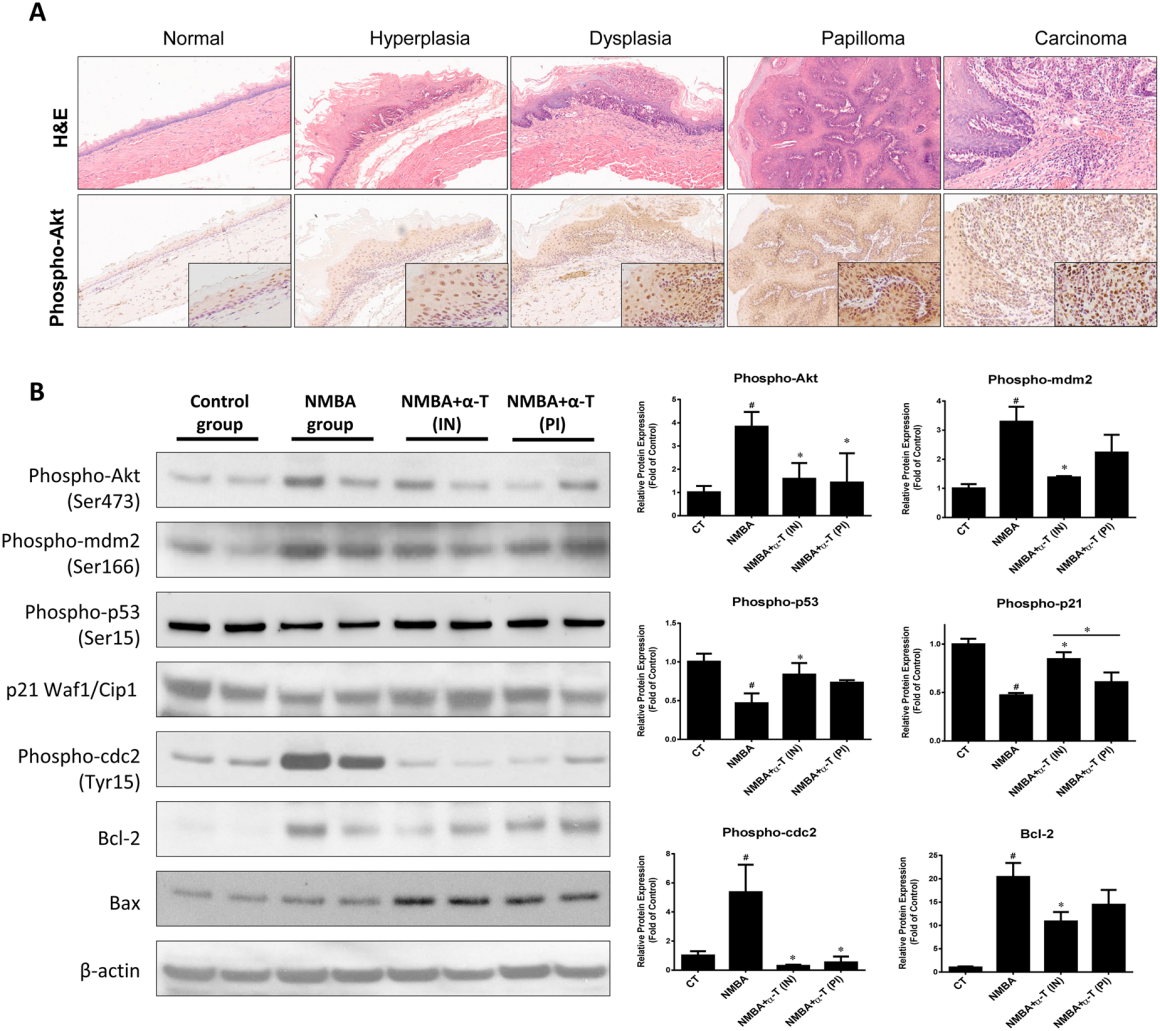
α-Tocopherol attenuated the activation of Akt signaling pathway in ESCC rat model **(A)** Immunostaining of Akt activation in pathological lesions of esophageal carcinogenesis. **(B)** Western blotting of Akt signaling pathway in rat esophagus. Two individual pooled samples from 12 rat of each group were analyzed. ^#^, p<0.05 compared with the control group; ^*^, p<0.05 compared with the NMBA group.

To further validate the role of α-Tocopherol in blocking Akt activation, we determined the relevant molecules in esophageal tissue from the ESCC rat model. In rat esophagus epithelium, the phosphorylation of Akt was significantly enhanced as the NMBA-induced pathological lesion gradually progressed (Figure 5A). Furthermore, the phosphorylation of Akt, mdm2, and cdc2 were consistently suppressed by supplementation with α-Tocopherol at the early stage when compared to the NMBA group (Figure 5B). On the contrary, the activation of p53 and p21 was facilitated by α-Tocopherol in response to Akt repression. Also, the anti-apoptotic protein Bcl-2 and the pro-apoptotic Bax were inversely regulated by α-Tocopherol, which is well correlated with the result from Het-1A cell model. Collectively, these *in vitro* and *in vivo* data suggest that α-Tocopherol inhibits NMBA-induced ESCC carcinogenesis by attenuating Akt activation, resulting in the subsequent suppression of cell growth and the promotion of apoptosis.

### PPARγ played an important role in the suppression of Akt signaling by α-Tocopherol

**Figure 6:**
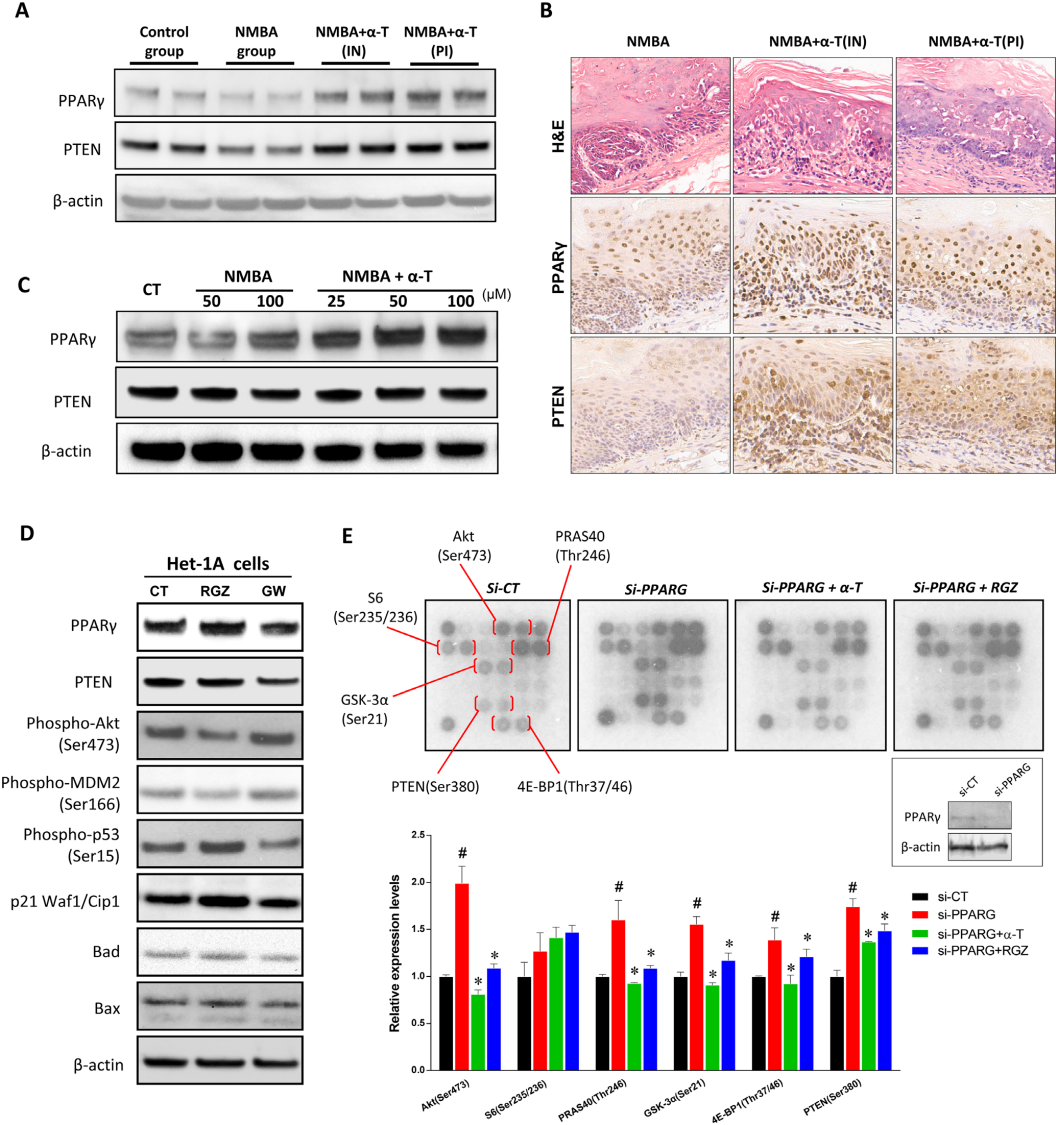
PPARγ played an important role in the suppression of Akt signaling by α-Tocopherol **(A)** Western blotting of PPARγ and PTEN in rat esophagus. Two individual pooled samples from 12 rat of each group were analyzed. **(B)** Immunostaining of PPARγ and PTEN in rat esophagus. **(C)** Western blotting of PPARγ and PTEN in Het-1A cells. Het-1A cells were treated with NMBA (50, 100 μM) with or without α-Tocopherol (25, 50, 100 μM) for 24h. **(D)** Western blotting of PPARγ and Akt pathway in Het-1A cells. Het-1A cells were treated with Rosiglitazone (RGZ, 20 μM) or GW9662 (GW, 20 μM) for 24h. **(E)** Proteomic array analysis on Akt signaling pathway. Het-1A cells were transfected with si-PPARG and then treated with α-Tocopherol (50 μM) or Rosiglitazone (RGZ, 20 μM) for 24h. Data are shown as mean ± standard deviation. ^#^, p<0.05 compared with the control group; ^*^, p<0.05 compared with the si-PPARG group.

Previous studies showed that γ- and δ-Tocopherol but not α-Tocopherol prevented mammary and prostate cancer by activating PPARγ in animal models [20, 21]. In this study, we investigated whether Akt activation in NMBA-induced esophageal carcinogenesis was inhibited by α-Tocopherol through regulating PPARγ. In ESCC rat model, the expression of PPARγ was apparently enhanced in animals supplemented with α-Tocopherol at the initiation stage and post-initiation stage. The tumor suppressor gene PTEN, which is transcriptionally activated by PPARγ and acts as a major negative regulator of the PI3K/Akt signaling, was also increased by α-Tocopherol (Figure 6A). Importantly, the initiation-stage supplementation resulted in more expression of PPARγ/PTEN in esophageal dysplasia when compared with the post-initiation stage supplementation (Figure 6B). In addition, α-Tocopherol also significantly elevated the expression levels of PPARγ and PTEN in the Het-1A cell model (Figure 6C).

To validate whether PPARγ activation by α-Tocopherol inhibits Akt signaling, the specific agonist Rosiglitazone (RGZ) and antagonist GW9662 were used in Het-1A cells. PPARγ activation by RGZ significantly inhibited the phosphorylation of Akt and mdm2, whereas the downstream p53, p21, Bad and Bax were inversely increased (Figure 6D). In contrast, PTEN and p53 were suppressed by GW9662. On the other hand, PPARγ knockdown with siRNA markedly increased the phosphorylation of Akt and its downstream target molecules (PRAS40, GSK-3α, and 4E-BP1), which was significantly inhibited by the presence of α-Tocopherol or RGZ (Figure 6E). Similarly, the phosphorylation of PTEN at Ser 380, which negatively regulates PTEN stability and may impair its biological activity, was increased by PPARγ repression but decreased by the presence of α-Tocopherol or RGZ. Taken together, these data suggested that PPARγ activation played a critical role in the suppression of Akt signaling pathway by α-Tocopherol.

### α-Tocopherol was not effective in activating PPARγ and deactivating Akt in esophageal cancerous cells

**Figure 7:**
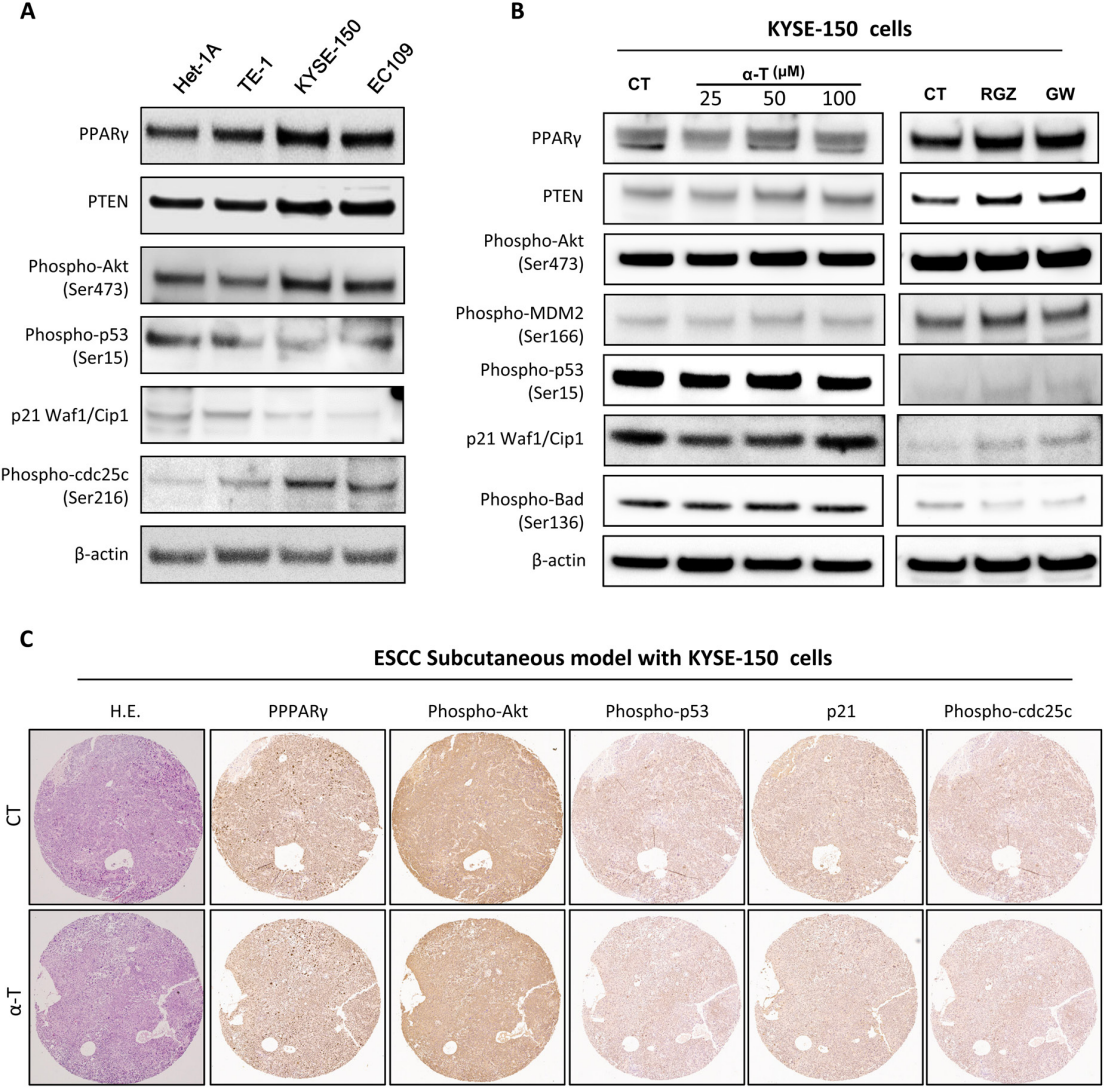
α-Tocopherol was not effective in activating PPARγ and deactivating Akt in esophageal cancerous cells **(A)** Endogenous levels of PPARγ, PTEN and Akt signaling molecules in normal cells (Het-1A) and malignant cell lines (TE-1, KYSE-150 and EC109). **(B)** Western blotting of PPARγ and Akt pathway in KYSE-150 cells. KYSE-150 cells were treated with α-Tocopherol (25, 50, 100 μM), Rosiglitazone (RGZ, 20 μM), or GW9662 (GW, 20 μM) for 24h. **(C)** Immunostaining of PPARγ and Akt pathway in ESCC Subcutaneous tumors generated with KYSE-150 cells.

We observed that α-Tocopherol was not effective in inhibiting cell proliferation in established tumors *in vivo* as well as in inducing cell cycle arrest and apoptosis in cancerous cells, here we also determined the effect of α-Tocopherol on PPARγ-Akt pathway in cancerous cells. In comparison with the Het-1A cells, Akt was constitutively activated in ESCC cell lines while p53 and p21were significantly suppressed (Figure 7A). Intriguingly, the basal levels of PPARγ and PTEN and were relatively higher in ESCC cell lines than in the normal Het-1A cells. In KYSE-150 cells, α-Tocopherol showed no impact on the PPARγ-Akt signaling pathway. Though RGZ and GW9662 slightly changed the expression of PPARγ and PTEN, but they did not significantly altered Akt activation (Figure 7B). In addition, the expression of PPARγ, phospho-Akt, phospho-53, p21, and phospho-cdc25C were not significantly altered by α-Tocopherol in the ESCC subcutaneous model with KYSE-150 cells (Figure 7C). Collectively, α-Tocopherol is not effective in activating PPARγ and deactivating Akt in esophageal cancerous cells.

## DISCUSSION

**Figure 8:**
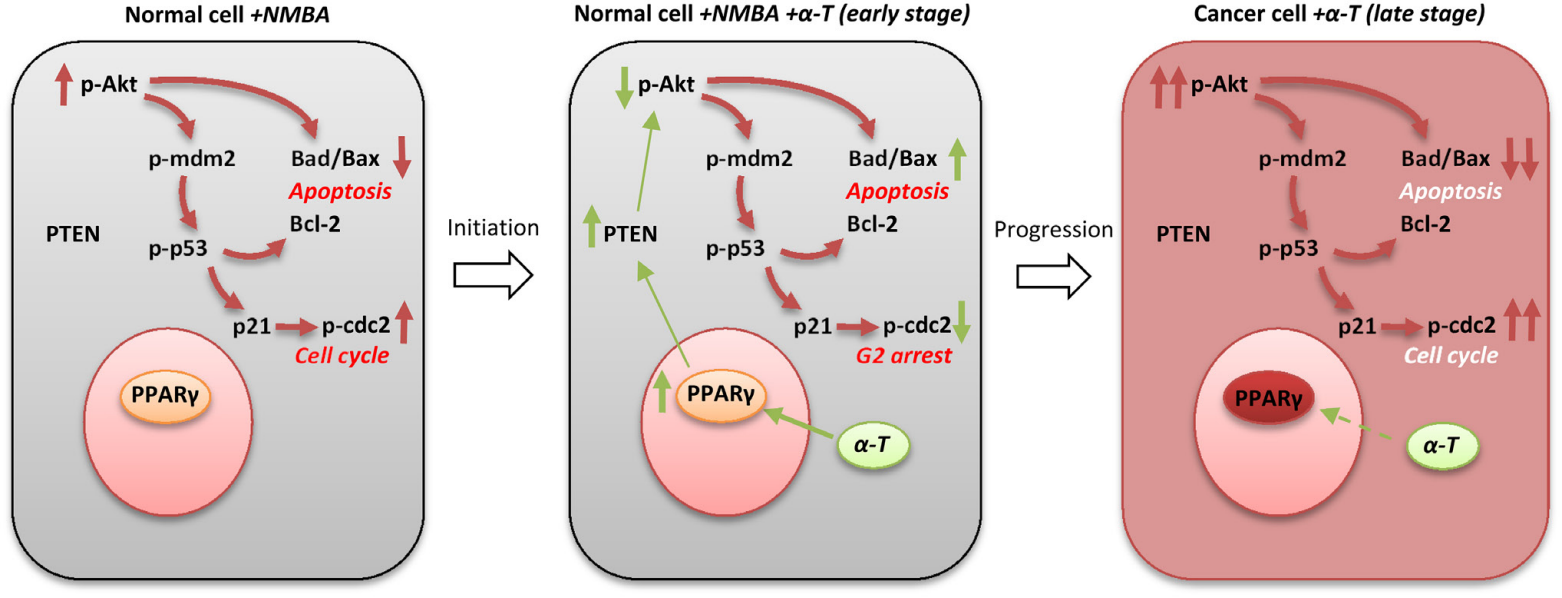
Schematic illustration of the chemopreventive effect of α-Tocopherol at the early stage of esophageal carcinogenesis (1) NMBA induces Akt activation in esophageal epithelium cells, resulting in the acceleration of cell cycle and the inhibition of apoptosis. (2) At the early stage of carcinogenesis, α-Tocopherol effectively increases the expression of PPARγ and PTEN, which attenuated the activation of Akt signaling pathway leading to cell cycle arrest and enhanced cell apoptosis. α-Tocopherol is effective in ESCC prevention during this stage. (3) As the pathologic lesions progressed (late stage), PPARγ and Akt become constitutively activated and insensitive to α-Tocopherol. Supplementation with α-Tocopherol at this stage is not effective for ESCC prevention.

Though laboratory research and clinical trials have highlighted the potential of using tocopherols in cancer chemoprevention, the preventive activities of tocopherols still remain controversial [5, 16]. Our previous studies revealed that early supplementation with α-Tocopherol and selenium had stronger preventive effect than late supplementation on esophageal carcinogenesis [18, 19]. In the present study, we showed that dietary supplementation with α-Tocopherol at the initiation-stage inhibited esophageal carcinogenesis in NMBA-induced ESCC rat model. Further studies in different experimental systems consistently demonstrated that PPARγ-Akt signaling pathway was regulated by α-Tocopherol and played a critical role during the entry stage of esophageal cancer (Figure 8).

Up to now, a number of studies have indicated the role of vitamin E involved in signal transduction that beyond its traditional antioxidant activity of scavenging reactive oxygen species (ROS) and reactive nitrogen species (RNS) [22]. Early investigation showed that inhibition of cell proliferation by tocopherols was correlated with the inhibition of protein kinase B (PKB/Akt) phosphorylation at Ser473 in human mastocytoma cell (HMC)-1 cells, whereas independent of oxidative stress [23]. In several other cancer cell lines, such as gastric cancer SGC-7901 cells [24], prostate cancer DU145 cells [25], myeloid leukemia U937 cells [26], or colon cancer HCT116 and HT29 cells [27], similar effects of tocopherols on Akt inhibition were observed. However, the influence of vitamin E on Akt signaling in esophageal cancer cells has not been systematically studied. Here, our data demonstrated that α-Tocopherol inhibited cell proliferation by suppressing Akt activation in ESCC rat model and in NMBA-treated Het-1A cell model. In particular, α-Tocopherol induced cells to undergo cell cycle G2-phase arrest. In agreement with previous studies, the blockade of cell cycle at G2-phase was closely associated with increased p21/Waf1 expression and reduced levels of inactivated phospho-cdc2 (Tyr 15) [28, 29].

In addition to cell proliferation, Akt plays an important role in apoptosis which offers a promising target for the therapy of human ESCC [30, 31]. It was reported that Vitamin E succinate down-regulated the constitutively active basal levels of Akt and decreased the phosphorylation of Akt substrates Bcl-2 associated death receptor in esophageal cancer EC109 cells [32]. In the Het-1A cell model, we also observed significant increase of apoptosis and expression of pro-apoptotic proteins (Bax and Bad) that induced by α-Tocopherol. However, little effect of α-Tocopherol on cell proliferation, cell cycle and apoptosis was found in the KYSE-150 and TE-1 cancer cells. One of the explanations to the discrepancy might be due to the analogue-specific activity of vitamin E (tocopherols, tocotrienols, succinates, etc.). For example, γ- and δ-tocotrienols had potent anti-proliferative activity and induced apoptosis through inhibition of PI3K/PKB in pancreatic cancer cells, but tocopherols were not able to induce the observed effects [33]. On the other hand, in aspect of the proliferation-inhibitory effects of tocopherols, ineffectiveness of the alpha forms of tocopherol was frequently observed in cancer cells [27, 34]. In esophageal cancer KYSE-150 and TE-1 cells, the effect of α-Tocopherol on cell growth *in vitro* and subcutaneous tumor growth *in vivo* was neither detected. The ineffectiveness of α-Tocopherol in established tumors or cancerous cell lines might be attributed to the constitutive Akt activation and the insensitive status to α-Tocopherol in malignant cells. The tissue- and cell-type selectivity of Akt activation in response to α-Tocopherol suggests more susceptive attenuation of esophageal carcinogenesis at the initiation stage than the later stage. These results confirmed the time-selective activity of α-Tocopherol in the chemoprevention of ESCC [19]. And this probably can explain that the preventive effect of supplementation with α-Tocopherol and selenium was only observed in subjects with mild esophageal dysplasia [35] or at younger ages [17].

As α-Tocopherol is not likely a competitive inhibitor for NMBA uptake or activity in esophageal cells (Supplementary Figure 1), the molecular mechanism underlying the time-selective effect of α-Tocopherol may be related to the Akt-upstream factors which dynamically change during carcinogenesis and meanwhile function differently in physiological or pathological condition, such as PPARγ [36]. Previous studies showed that PPARγ can suppress beta-catenin levels and colon carcinogenesis but only before damage to the APC/beta-catenin pathway, suggesting a potentially important use for PPARγ ligands as chemopreventive agents in colon cancer [37, 38]. Increasing evidence suggests that PPARγ activity is attenuated during the transition from adenoma to adenocarcinoma, likely explaining why PPARγ agonists are effective in blocking the early stages of tumorigenesis while no effect is detected in advanced tumor stages [39, 40]. In addition, small clinical trials showed no beneficial effects of conventional PPARγ ligands in cancer patients [41, 42], supporting the idea that PPARγ agonists should be used as chemopreventive agents other than therapeutic drugs.

Recent studies demonstrated that δ- and γ-tocopherols inhibited mammary tumorigenesis by increasing expression of PPARγ and its downstream gene PTEN, and as a result inhibiting the Akt-mediated cell survival pathway [43, 44]. It has been established that PTEN as the tumor suppressor is essential for regulating the highly oncogenic pro-survival PI3K/AKT signaling [45]. In agreement, our study demonstrated that α-Tocopherol significantly increased the expression of PPARγ and PTEN in rat esophageal epithelium as well as in Het-1A cells, which was inversely correlated with Akt activation. Upregulation of PPARγ expression by α-Tocopherol might be through indirect mechanisms [22]. As α-Tocopherol shares structural similarity to the known PPARγ activator troglitazone, α-Tocopherol might bind to and activate small amount of PPARγ which in turn binds to cis-elements in the promoter region of the PPARγ gene leading to induction of transcription and protein levels [46]. On the other hand, α-Tocopherol might be able to keep higher levels of PPARγ possibly by preventing and/or by postponing the degradation events regulated by the proteasome pathway [47]. Furthermore, the knockdown of PPARγ with RNA interference and in combination with α-Tocopherol or PPARγ agonist RGZ consistently suggested that the inhibition of Akt by α-Tocopherol was, at least in part, dependent on PPARγ activation.

Though some small molecule agonist of PPARγ has been shown to reduce the phosphorylation of Akt at Ser473 and exert therapeutic effects in esophageal cancer, the relation between PPARγ protein expression and prognosis in human ESCC still remains unclear [48]. As increased activation of the lipogenesis pathway is a hallmark of cancer cells, PPARγ is ubiquitously expressed in many cancers and promotes lipid metabolism, glucose homoeostasis and tumor progression. In this study, we observed relatively higher basal level of PPARγ and PTEN in ESCC cell lines when compared with the normal Het-1A cells. The heterogeneous expression of PPARγ may be responsible for the insusceptibility of Akt signaling to α-Tocopherol in malignant cells (Figure 8). Further studies on the potential role of PPARγ as chemopreventive or therapeutic target for ESCC are warranted [49].

In conclusion, this study demonstrated the tumor-suppressive effect of α-Tocopherol and underlying mechanism involving inhibition of Akt pathway. We showed that early-stage supplementation with α-Tocopherol significantly inhibited cell proliferation and promoted apoptosis. We further demonstrated that α-Tocopherol attenuated the activation of Akt signaling pathway through modulating PPARγ. This study suggested that dietary supplementation with α-Tocopherol could be served as a potential strategy for ESCC chemoprevention.

## MATERIALS AND METHODS

### Reagents

N-nitrosomethylbenzylamine (NMBA) was purchased from Ash Stevens Inc. (Detroit, MI, USA). α-Tocopherol (α-T) with purities of 95.5% was purchased from Sigma Aldrich (St. Louis, MO, USA). *in vitro* studies, α-Tocopherol was purified by flash chromatography to purities > 99%. Primary antibodies against PCNA, PPARγ, PTEN, phospho-Akt (Ser473), phospho-mdm2 (Ser166), phospho-p53 (Ser15), p21 Waf1/Cip1, phospho-Bad (Ser136), Bax, Bcl-2, phospho-cdc2, phospho-cdc25C, and HRP-labeled secondary antibodies were purchased from Cell Signaling Technology (Danvers, MA, USA). Antibodies for immunohistochemistry against phospho-Akt, PPARγ, and p53 were purchased from Merck KGaA (Darmstadt, Germany) or LifeSpan BioSciences. Inc. (Seattle, USA). The HRP-labeled anti-β-actin antibody was from Sigma-Aldrich (St. Louis, MO, USA). The PPARG-specific agonist Rosiglitazone (RGZ) and antagonist GW9662 (GW) were purchased from Selleck Chemicals (Houston, TX, USA).

### Animal diet

Two different diets were prepared by Keaoxieli Diet Co. (Beijing, China): the normal diet and the alpha-Tocopherol (α-T) enriched diet. The normal diet was produced according to AIN-93M formula which contained 80 IU/kg α-Tocopherol [50]. The α-T enriched diet was prepared with the same formula but contained 162 IU α-Tocopherol per kg diet. The purpose was to mimic the Vitamin E intervention in the Linxian trial [8].

### ESCC rat model

The ESCC rat model was constructed according to the method previously established in our lab [18, 19]. Six-week-old male F344 rats were purchased from Vitalriver (Beijing, China) and randomly distributed into four groups. Animals were housed in a controlled environment with a 12 h light/dark cycle, and given water ad libitum. As shown in Figure 1A, three groups of rats were subcutaneously injected with NMBA at 0.35 mg/kg body weight 3 times per week for 5 weeks, and maintained on the specific diets: the normal diet (group NMBA), α-T enriched diet at the initiation stage (Week 0-5) (group NMBA+α-T(IN)), α-T enriched diet at the post-initiation stage (Week 6-10) (group NMBA+α-T(PI)), respectively. The control group (group CT) were treated with the solvent vehicle (20% DMSO/water) and given the normal diet throughout the entire experiment (Week 0-25). All the animals were acclimatized with the corresponding diets for 2 weeks before NMBA treatments, body weight and food intake was measured once a week during the experiment. The levels of alpha-Tocopherol in animal plasma were monitored at Week 0, 5, 10 and 25 using High-performance liquid chromatography (HPLC). All animal experiments were followed the protocol (#2014013) approved by the Institutional Animal Care and Use Committee of China National Center for Food Safety Risk Assessment (CFSA).

### Tumor incidence and pathological analysis

At the end of Week 25, rats were humanely sacrificed by pentobarbital anesthetization 2 hours after injection of 5-bromo-2-deoxyuridine (BrdU, 50 mg/kg, i.p.). The esophagi were excised and opened longitudinally, and visible tumors (≥1mm in diameter) were counted. Then the esophagus was cut longitudinally, with one-half fixed in 10% neutral buffered formalin for pathological analysis. The other half of the esophagus was stripped of muscle, quickly frozen in liquid nitrogen and then stored at -80°C for later use. H&E stained sections were used for histological diagnosis of normal, hyperplasia, dysplasia, papilloma and carcinoma according to previous diagnostic criteria [51].

### Cell culture and treatments

The immortalized human normal esophageal epithelium cells Het-1A (CRL-2692) were purchased from American Type Culture Collection (ATCC, VA, USA) and cultured in Bronchial epithelial cell basal medium (BEGM) with all the additives (Lonza, MD, USA). Human ESCC cells lines KYSE-150 and TE-1 were obtained from the Cell Bank of Shanghai Institutes for Biological Sciences (Chinese Academy of Sciences, Shanghai, China). KYSE-150 cells were cultured in mixture medium of RPMI-1640/F12 (v/v=1:1), and TE-1 cells were cultured in RPMI-1640 medium, all mediums were supplemented with 10% fetal bovine serum, penicillin (100 μg/ml) and streptomycin (100 μg/ml). Cells were seeded at concentration of 1×10^5^/ml in multi-well plates for 24 h and then treated with NMBA, α-T or their combination for certain periods of time before collecting for analysis.

### ESCC subcutaneous model

BALB/c athymic nude mice were obtained from Vitalriver (Beijing, China) and randomized into two groups which were respectively fed on the normal diet (group CT), or α-T enriched diet (group α-T). To establish the subcutaneous ESCC model, cultured KYSE-150 or TE-1 cells were collected after trypsin digestion and resuspended with fresh medium and Matrigel Basement Membrane Matrix (BD, Franklin Lakes, NJ, USA), and then 10^6^ cells in 0.1ml were subcutaneously injected into the back of mice. Tumor volume was measured with caliper in two perpendicular diameters of the implant and calculated using formula ½(*a×b*^2^), where “*a*” is the long diameter and “*b*” is the short diameter. Tumor volume was determined once every 5 days and recorded for 20 days until animal sacrifice. The subcutaneous tumors were fixed in 10% neutral buffered formalin for immunostaining.

### Cell proliferation, cell cycle and apoptosis

Cells seeded in multi-well plates were treated with NMBA (100μM), α-Tocopherol (25, 50, 100μM), or their combination for 48-72h. Cell proliferation was determined with Cell Counting Kit-8 (CCK-8) (Sigma). For cell cycle analysis, cells were collected and fixed with 70% ethanol at 4°C for 4h. After washing with cold PBS, cells were resuspended with 500μl of propidium iodide (PI) (Life Technology, Oregon, USA) and incubated at 37°C in dark for 20 minutes followed by analysis with FACSCalibur flow cytometer (BD Biosciences, San Jose, CA, USA). Cell apoptosis was determined with the Annexin V-FITC Apoptosis Detection Kit (BD Biosciences, San Jose, CA, USA) according to the instruction of manufacturer.

### Transcriptome microarray

Het-1A cells were treated with NMBA (100 μM) for 5 weeks. Total RNA was extracted from Het-1A cells using Trizol reagent (Invitrogen, Carlsbad, CA, USA), and purified with magnetic beads of Agencourt Ampure (APN 000132, Beckman Coulter). The GeneChips of Human Transcriptome Array 2.0 (Affymetrix, USA) were used for whole-transcript expression profiling. The genes with threshold values of fold change ≥1.5 or ≤−1.5 were designated as differentially expressed. The Database for Annotation, Visualization and Integrated Discovery (DAVID) was used to determine pathways and processes of major biological significance and importance based on the Gene Ontology (GO) annotation function and Kyoto Encyclopedia of Genes and Genomes (KEGG) pathway function.

### Small interfering RNA (siRNA) and transfection

The Trilencer-27 human PPARG siRNA (si-PPARG) and Trilencer-27 universal scrambled negative control siRNA (si-CT) were purchased from OriGene Technologies, Inc. (Rockville, MD, USA). Het-1A cells seeded in multi-well plates were transfected with siRNA at a 10 nM final concentration using siTran 1.0 following the manufacturer's protocol. After 24h, cells were treated with α-Tocopherol (50 μM), or Rosiglitazone (RGZ, 20 μM) for another 24h. Subsequently, cell lysates were collected for PathScan Akt Signaling Array assay according to the instruction provided by manufacture (Cell Signaling Technology Inc., Danvers, MA, USA).

### Western blotting

Homogenated proteins (10-40 μg) from esophageal tissue or cell lysate were resolved on SDS-polyacrylamide gels (NuPAGE, Invitrogen, Carlsbad, CA, USA), and electrophoretically transferred onto Polyvinylidenefluoride membranes. After blocking with 5% skim milk, the membranes were incubated with specific primary antibodies and then corresponding HRP-labeled secondary antibodies. Membranes were developed with West Femto Chemiluminescent substrate (Thermo, Rockford, IL, USA) and the images were captured with Syngene GeneGnome XRQ system (BioTek, HK, China). The relative expression of proteins were quantified by bands area and density with Image J software and normalized with β-actin.

### Immunohistochemistry

For paraffin embedded tissue section, antigens were unmasked in citrate buffer for 10 min at 90°C. Endogenous peroxidase was quenched by 10 min incubation in 3% hydrogen peroxide in PBS. After blocking with 5% normal goat serum, the sections were incubated with primary antibody overnight at 4°C. Then HRP-conjugated secondary antibody (Cell signaling Technology Inc., Danvers, MA, USA) and diaminobenzidine (DAB) were applied to visualize the expression and localization of antigens. BrdU immunohistochemistry was performed using an *in-situ* detection kit (BD Biosciences, Franklin Lakes, NJ, USA) according to the instruction.

### Statistical analysis

Incidences of visible tumors, microscopic papilloma and carcinoma were compared using χ^2^ and Fisher's exact test. Tumor multiplicity, numbers of histopathological lesions, levels of protein expression were expressed as mean ± standard deviation and analyzed using one-way Analysis of Variance (ANOVA). Turkey-Kramer test was used for multiple comparisons. P value <0.05 were considered statistically significant.

## SUPPLEMENTARY MATERIALS FIGURES AND TABLES


