# Supplementary material for: Alpha-Tocopherol prevents esophageal squamous cell carcinoma by modulating PPARγ-Akt signaling pathway at the early stage of carcinogenesis
Source: Oncotarget. 2017 Sep 30;8(56):95914–30. doi: 10.18632/oncotarget.21437 (PMC5707070; doi:10.18632/oncotarget.21437)
Supplement: Supplementary file 1 [file oncotarget-08-95914-s001.pdf]

Alpha-Tocopherol prevents esophageal squamous cell carcinoma by modulating PPARγ-Akt signaling pathway at the early stage of carcinogenesis

SUPPLEMENTARY MATERIALS

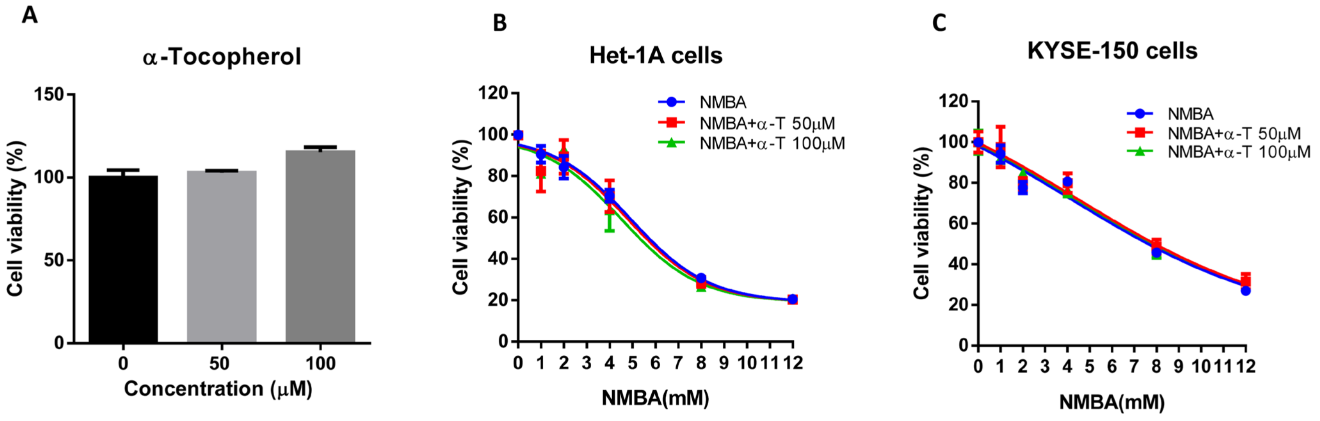

Supplementary Figure 1: α-Tocopherol is not a competitive inhibitor for NMBA activity in esophageal cells. (A) α-Tocopherol did not induce cytotoxicity in Het-1A cells. **(B)** α-Tocopherol (50 and 100 μM) did not affect the toxicity of NMBA in Het-1A cells. **(C)** α-Tocopherol did not affect the toxicity of NMBA in KYSE-150 cells. Cells were treated with NMBA in the absence or presence of α-Tocopherol for 24 h, cell viability was determined with Cell counting kit (CCK-8).

Supplementary Table 1: Pathway enrichment and identified genes

Pathways	Genes
PI3K-Akt signaling pathway	AKT3, CCNE2, CHUK, FN1, IL7, IL7R, ITGA4, ITGAV, ITGB6, LAMC2, TNC, VEGFC
Pathways in cancer	AKT3, BIRC3, CCNE2, CHUK, FN1, FZD3, IL8, ITGAV, LAMC2, TGFBR1, VEGFC
Cytokine-cytokine receptor interaction	CCL2, IL23A, IL6ST, IL7, IL7R, IL8, INHBA, TGFBR1, TNFSF18, VEGFC
Focal adhesion	AKT3, BIRC3, FN1, ITGA4, ITGAV, ITGB6, LAMC2, TNC, VEGFC
Small cell lung cancer	AKT3, BIRC3, CCNE2, CHUK, FN1, ITGAV, LAMC2
ECM-receptor interaction	CD44, FN1, ITGA4, ITGAV, ITGB6, LAMC2, TNC
Chagas diseases	AKT3, C3, CCL2, CHUK, IL8, TGFBR1, TLR6
NOD-like receptor signaling pathway	BIRC3, CCL2, CHUK, IL8, TNFAIP3
Arrhythmogenic right ventricular cardiomyopathy (ARVC)	CDH2, ITGA4, ITGAV, ITGB6, RYR2
Taste transduction	PRKACB, TAS2R14, TAS2R30, TAS2R31
